# Satisfaction and Cost-Related Access Barriers Among American Indian and Alaska Natives Adults by Insurance Type

**DOI:** 10.1007/s11606-022-07830-9

**Published:** 2022-10-17

**Authors:** Tristan Moss-Vazquez, Charlie M. Wray, Lenny Lopez, Meena Khare, Salomeh Keyhani

**Affiliations:** 1 Northern California Institute for Research and Education, San Francisco Veterans Affairs Medical Center, San Francisco, USA; 2 Department of Medicine, University of California, San Francisco, San Francisco, CA USA; 3 Division of Hospital Medicine, San Francisco Veterans Affairs Medical Center, San Francisco, CA USA; 4 Division of General Internal Medicine, San Francisco Veterans Affairs Medical Center, San Francisco, CA USA

## INTRODUCTION

American Indian/Alaska Native (AIAN) adults face structural barriers such as inadequate education, disproportionate poverty, discrimination in the delivery of health services, and cultural differences. The result is higher rates of poor health, obesity, hypertension, and diabetes compared to the general population.^1^ Given their worse health and greater care needs, they may experience more access and cost-related access barriers.^2^ Although some AIAN individuals receive health care via the Indian Health Service (IHS), many receive care in the private sector via other forms of insurance coverage. Research conducted over two decades ago demonstrated that AIANs covered solely by IHS had better access to care than AIANs with no coverage.^3^ In comparison to AIANs with other forms of coverage, AIANs with IHS were less likely to have unmet medication needs.^3^ Using recent data, we compared cost-related access barriers and satisfaction with care among AIAN individuals covered by the IHS to those covered by other forms of insurance.

## METHODS

Using pooled data from 2016–2018 Behavior Risk Factor Surveillance (BRFSS), we identified all individuals who self-identified as AIAN among a random sample of adults in 17 states and DC.^4^ To assess coverage, we used the question, “What is the primary source of your health care coverage?” Of 2699 AIAN individuals who responded, we excluded 13 individuals who had reported no insurance coverage resulting in an analytic sample of 2686 AIAN adults representing about 700,000 AIAN adults. Survey details are available on the BRFSS website.^4^

We examined two domains among this sample: self-reported cost-related access barriers and satisfaction with care. Experience with cost-related access barriers was assessed by the following questions: (1) “Was there a time in the past 12 months when you needed to see a physician but could not because of cost?” (2) “In the past 12 months was there a time when you did not take your prescription medications due to cost?” (3) “Do you currently have any health care bills that are being paid over time?” Satisfaction was assessed with the question, “In general, how satisfied are you with the care you received?”

We examined the baseline characteristics of the respondents and then performed multivariable analyses, controlling for age, gender, and baseline health, comparing AIAN adults with IHS to other forms of insurance coverage (Medicare, Medicaid, VA/Military, private employer/individual, other insurance). The analyses accounted for non-responses and the complex survey design.

## RESULTS

Among the 2686 AIAN adult respondents, 640 were covered by IHS and 2046 had other forms of insurance. Adults covered by IHS were younger (median age 37.7 (±2.3) versus 46.6 (±1.1) years) and reported better health compared to those with other coverage (Table 1).

**Table 1:** Baseline Comparison of Sociodemographic Characteristics Among US AIAN Adults with Different Types of Health Care Coverage

	AIAN who get health insurance coverage via IHS	AIAN with non-IHS health insurance coverage^b^	*P*-value
Number of respondents	*N*=640	*N*=2046	
Median age (±SD)	37.7 (2.3)	46.6 (1.04)	
Weighted %			
Age			
18 to 44	64.1	45.7	<0.001
45 to 64	26.0	36.9	
65+	9.8	17.3	
Female	48.5	51.4	0.60
Married	32.7	43.0	0.04
Education			
Less than high school	10.9	16.4	0.11
High school graduate	43.4	34.6	
Some college	34.7	33.2	
Completed college	11.0	15.7	
Employed	56.2	52.8	0.53
Annual household income			
<$10,000	5.4	11.8	0.002
$10,000 to $19,999	29.4	19.0	
$20,000 to $34,999	31.2	23.8	
$35,000 to $49,999	12.7	14.1	
$50,000 to $74,999	11.8	12.8	
≥$75,000	9.5	18.6	
General health			
Excellent	17.0	13.3	0.009
Very good	25.8	23.1	
Good	40.1	32.4	
Fair	11.7	22.3	
Poor	5.3	8.8	
Comorbidities			
Obesity (BMI>30)	41.8	38.2	0.51
Coronary artery disease	5.8	5.7	0.97
COPD	7.4	13.8	0.02
Diabetes	16.2	16.6	0.91
Has smoked 100+ cigarettes	52.1	49.6	0.65
Functional status			
Difficulty in concentrating or remembering	16.8	22.0	0.25
Difficulty in walking or climbing stairs	9.7	24.4	<0.001
Difficulty in doing errands alone	7.7	13.1	0.06
Difficulty seeing a doctor due to cost	16.6	15.2	0.74
Did not take medications due to costs	5.1	13.4	<0.001
Have outstanding medical debt	19.4	25.7	0.19
Overall satisfaction with health care	50	56.2	0.25

Abbreviations: *SD*, standard deviation; *BMI*, body mass index (calculated as weight in kilograms divided by height in meters squared); *COPD*, chronic obstructive pulmonary disease

^a^Data are presented as weighted percentages of individuals unless otherwise indicated. States covered in this study include Delaware, Florida, Georgia, Kentucky, Louisiana, Maine, Minnesota, Mississippi, Nebraska, New Hampshire, New Jersey, New Mexico, Oklahoma, Oregon, Pennsylvania, Tennessee, Wisconsin, and the District of Columbia

^b^Non-IHS health insurance includes employer sponsored, individually purchased, Medicare, Medicaid, and Veterans Health Administration or military

Table 1 demonstrates that AIAN adults who had coverage through the IHS less commonly reported not taking medications due to costs (5.1% vs. 13.4%, *P*<0.001). There was no significant difference in other outcomes. Multivariable analyses demonstrated that AIANs with IHS were less likely to report not taking medications due to costs in comparison to AIANs with other coverage (odds ratio (OR), 0.39; 95% CI, 0.20–0.75). No differences were found in difficulty seeing a physician because of cost (OR, 1.28; 95% CI, 0.72–2.27), medical debt (OR, 0.70; 95% CI, 0.39–1.25), or satisfaction with care (OR, 0.76; 95% CI, 0.51–1.15) between AIAN individuals with IHS and other coverages (Table 2).

**Table 2:** The Association of Insurance Type with Cost-Related Access Barriers and Satisfaction with Care Among American Indian and Alaska Natives Adults

	Adjusted odds ratios* (95% CI)	*P*-value
	*N*=640	
Difficulty seeing a doctor due to cost	1.28 (0.72–2.27)	0.40
Did not take medications due to costs	0.39 (0.20–0.75)	0.005
Have outstanding medical debt	0.70 (0.39–1.25)	0.23
Overall satisfaction with health care	0.76 (0.51–1.15)	0.20

*Adjusted for age, sex, and general health

## DISCUSSION

Prior studies have demonstrated that cost is an important driver of health care utilization and medication adherence with detrimental downstream effects on health outcomes.^5^ We found that AIAN adults with IHS coverage were less likely to report cost-related medication adherence issues compared to other types of insurance. Higher patient satisfaction scores are associated with better health outcomes, adherence, and lower utilization.^6^ AIAN satisfaction was low overall across insurance types with no significant difference by coverage type suggesting there may be other access and quality barriers that were not measured in this study. Several limitations deserve comment: (1) we cannot account for all unmeasured confounding of assignment to IHS and (2) the survey excluded several states with significant AIAN populations (e.g., Arizona, South Dakota). The IHS delivers care to approximately 2.2 million individuals. Consistent with prior research, this study suggests that the IHS continues to play an important role in eliminating cost-related access barriers among AIAN populations.^3^
